# Supplementary material for: Influence of Metal Ions on Model Protoamphiphilic Vesicular Systems: Insights from Laboratory and Analogue Studies
Source: Life (Basel). 2021 Dec 16;11(12):1413. doi: 10.3390/life11121413 (PMC8708898; doi:10.3390/life11121413)
Supplement: Supplementary file 1 [file life-11-01413-s001.zip › life-1500974-supplementary.pdf]

Supplementary Information

Influence of Metal Ions on Model Protoamphiphilic Vesicular Systems: Insights from Laboratory and Analogue Studies

Manesh Prakash Joshi [1,*], Luke Steller [2], Martin J. Van Kranendonk [2] and Sudha Rajamani [1,*]

[1] Department of Biology, Indian Institute of Science Education and Research, Dr. Homi Bhabha Road, Pune 411008, Maharashtra, India
[2] Australian Centre for Astrobiology, and School of Biological, Earth and Environmental Sciences, University of New South Wales, Kensington, NSW 2052, Australia; l.steller@unsw.edu.au (L.S.); m.vankranendonk@unsw.edu.au (M.J.V.K.)
* Correspondence: manesh.joshi@students.iiserpune.ac.in (M.P.J.); srajamani@iiserpune.ac.in (S.R.); Tel.: +91-20-2590-8061 (S.R.)

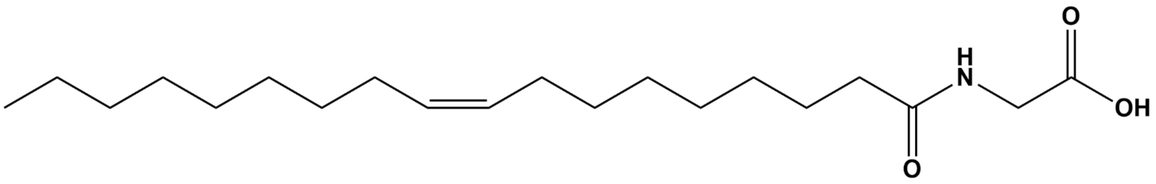

N-oleoyl glycine (NOG)

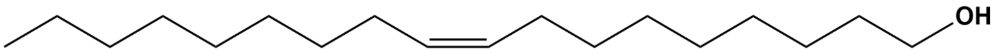

Oleyl alcohol (OOH)

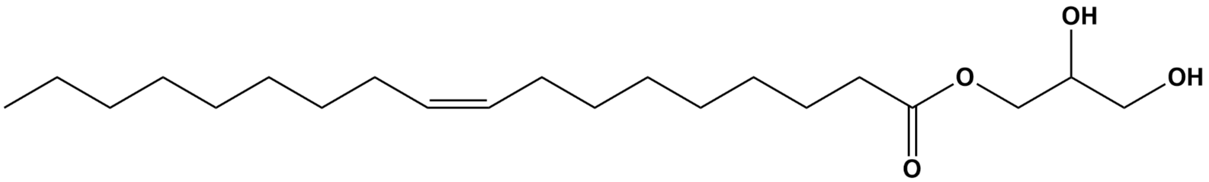

Glycerol 1-monooleate (GMO)

Figure S1. Chemical structures of different amphiphiles used in this study along with their abbreviations. NOG has been shown in its protonated form.

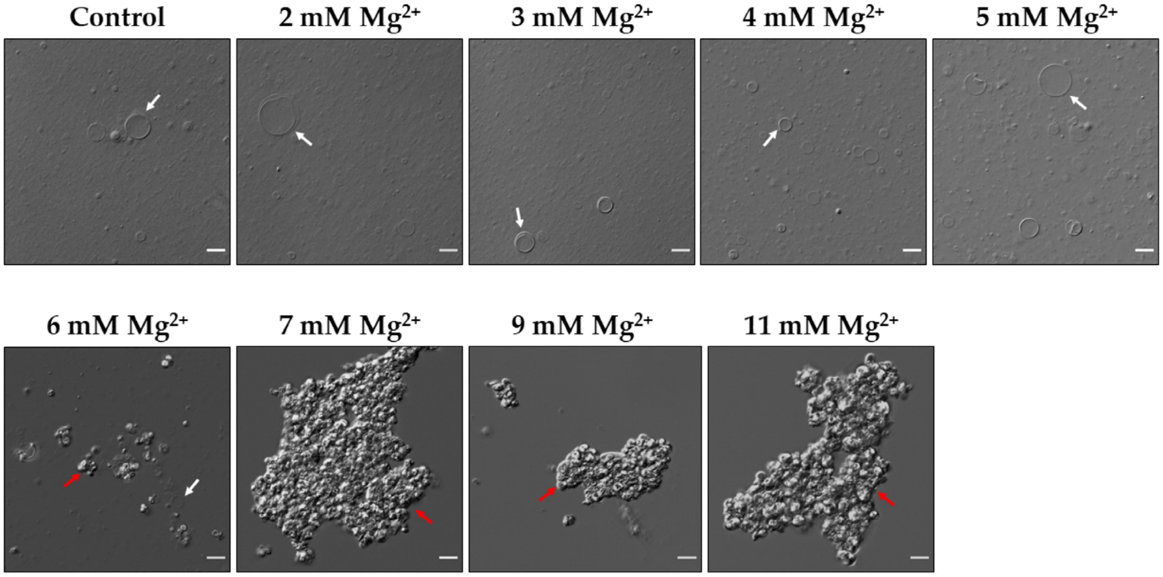

Figure S2. Effect of Mg^{2+} on the stability of pure NOG vesicles. Vesicles are stable up to Mg^{2+} concentrations of 5 mM. At 6 mM Mg^{2+}, small magnesium-induced aggregates start to appear in the solution, although free vesicles are also present along with these aggregates. However, from 7 mM Mg^{2+} onwards, only large-sized aggregates are present in the solution. Vesicles and aggregates are indicated by white and red arrows, respectively. NOG concentration is 6 mM. Scale bar is 10 μm.

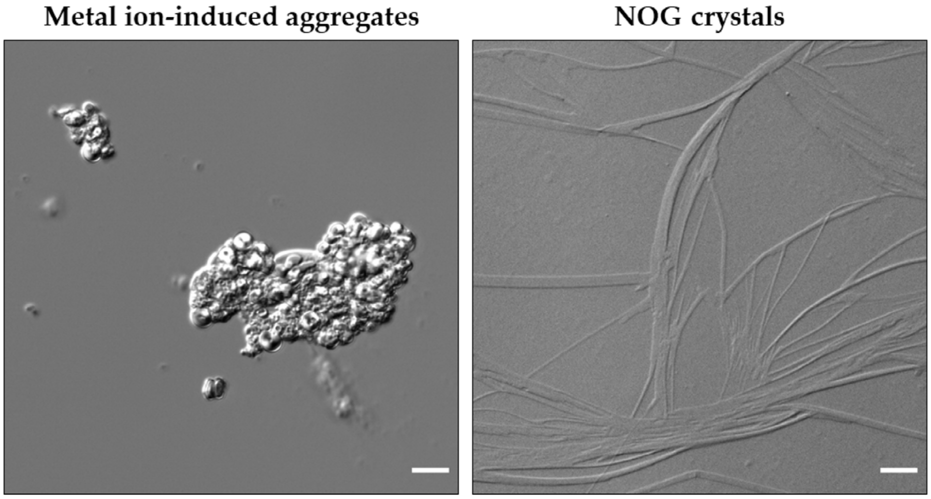

Figure S3. The morphology of metal ion-induced aggregates of NOG is different from that of NOG crystals. NOG crystals usually form at a lower temperature like 25 °C. Scale bar is 10 μm.

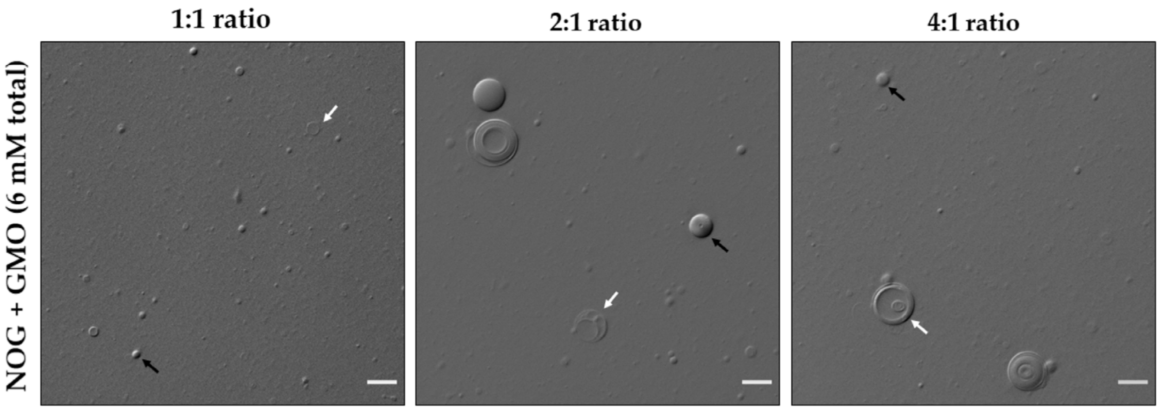

Figure S4: A mixed system of NOG and GMO forms a heterogeneous population of vesicles and droplets irrespective of the NOG to GMO ratio used. The vesicles and droplets are indicated by white and black arrows respectively. Total amphiphile concentration is 6 mM. Note that the size of both vesicles and droplets is smaller in the reaction containing NOG to GMO in 1:1 ratio when compared to other two reactions. Scale bar is 10 µm.

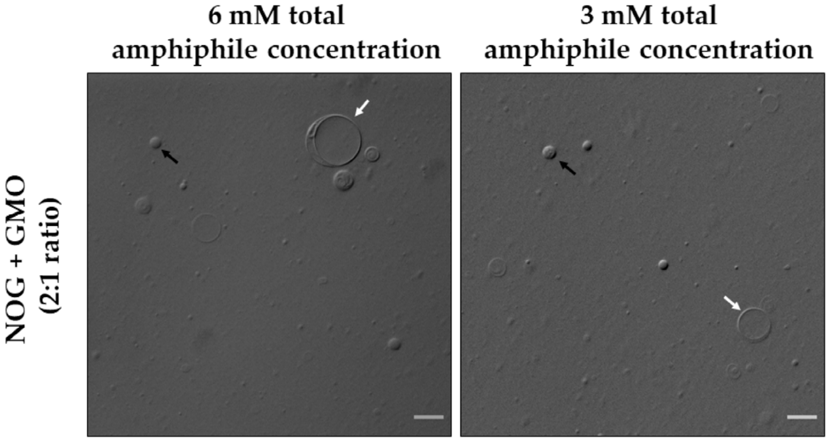

Figure S5: NOG + GMO (2:1) mixed system behaves similarly at different total amphiphile concentrations. Above panels show this behavior at 6 mM and 3 mM concentration, where a mixture of vesicles and droplets (indicated by white and black arrows respectively) is observed. Scale bar is 10 µm.

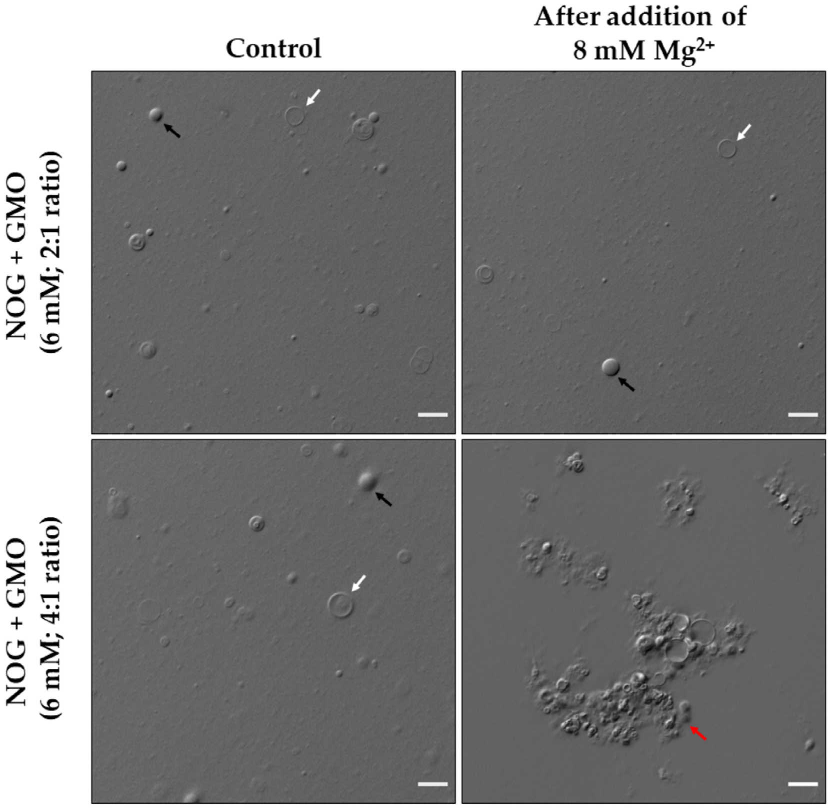

Figure S6: Decreasing the GMO concentration in the NOG + GMO mixed system also decreases the overall Mg^{2+} stability of the system. NOG + GMO (2:1 ratio) system retains free vesicles and droplets in the presence of 8 mM Mg^{2+}, which is just at the beginning of the transition phase. However, at the same magnesium concentration, NOG + GMO (4:1 ratio) system collapses into large-sized aggregates, which indicates that it has already reached an aggregate phase. Vesicles, droplets and aggregates are indicated by white, black and red arrows respectively. Scale bar is 10 μm.

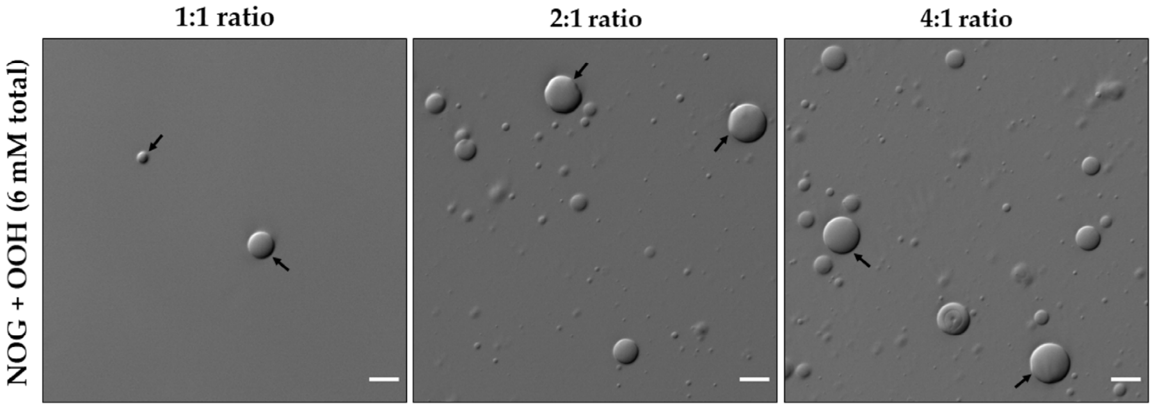

Figure S7: NOG + OOH mixed system predominantly forms droplets irrespective of the NOG to OOH ratio. Droplets are indicated by black arrows. The increasing number of droplets with increasing NOG to OOH ratio potentially reflects the differential solubility of these systems in water. Scale bar is 10 μm.

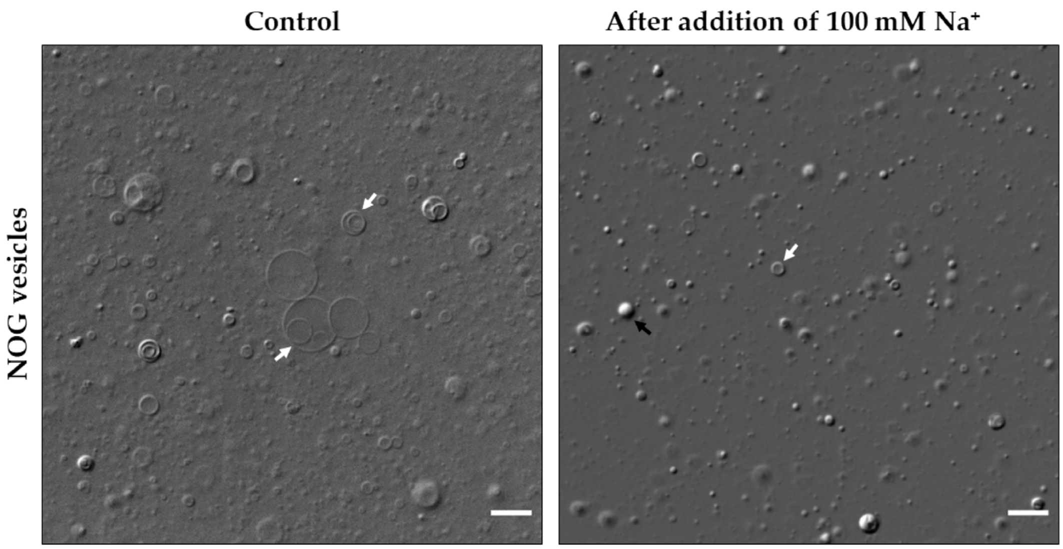

Figure S8: Effect of NaCl addition on pure NOG vesicles. The external addition of 100 mM NaCl to the solution containing preformed NOG vesicles results in the shrinkage of the vesicles and also the formation of small-sized droplets. Vesicles and droplets are indicated by white and black arrows respectively. NOG concentration is 6 mM. Scale bar is 10 μm.

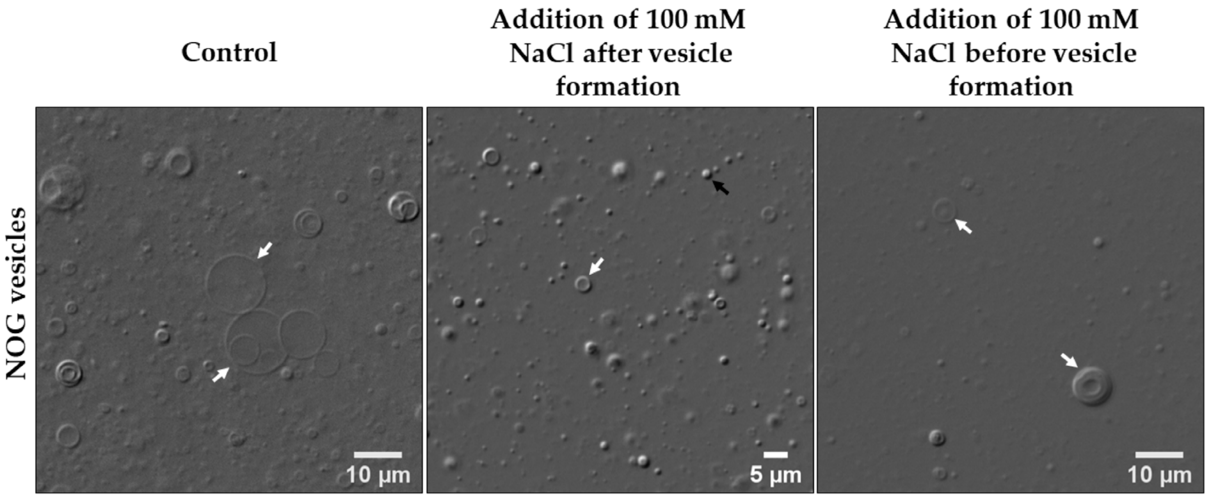

Figure S9: NOG vesicle formation with increased Na^{+} concentration in the buffer. The vesicle shrinkage and droplet formation effect was not very prominent when 100 mM NaCl was added in the acetate buffer before the vesicles were formed (rightmost image). The (rescaled) control and the external addition of 100 mM NaCl reaction images from Figure S8 have been included for comparison. . Vesicles and droplets are indicated by white and black arrows respectively. NOG concentration is 6 mM.

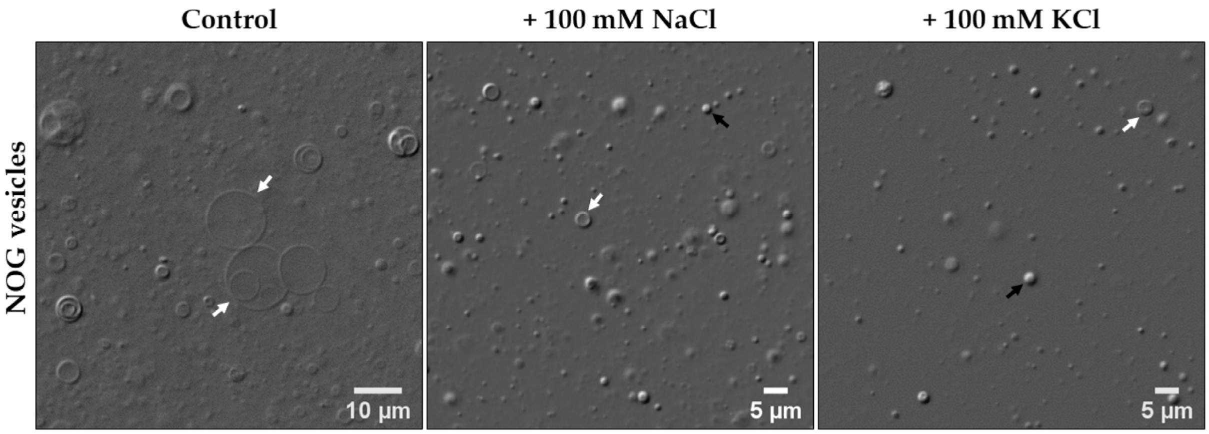

Figure S10: Effect of K^+ on the stability of pure NOG vesicles. The vesicle shrinkage and droplet formation phenomenon was also observed upon the external addition of KCl to preformed NOG vesicles (rightmost image), similar to that of the NaCl addition. The (rescaled) control and the 100 mM NaCl added reaction images from Figure S8 have been included for comparison. Vesicles and droplets are indicated by white and black arrows, respectively. NOG concentration is 6 mM.

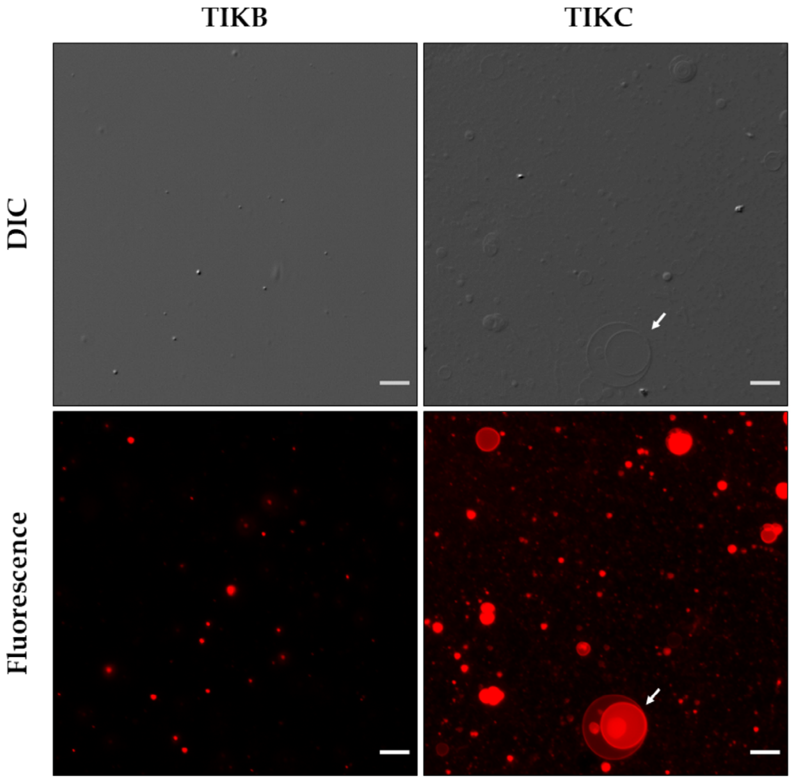

Figure S11: Vesicle formation behavior of NOG + OOH mixed system in hot spring water samples. NOG + OOH (6 mM; 2:1 ratio) system readily formed vesicles (indicated by white arrows) in TIKC (right panel), but not in TIKB (left panel) water. Vesicles were imaged by both DIC and fluorescence microscopy. For the fluorescence imaging, vesicles were stained with an amphiphilic dye named octadecyl rhodamine-B chloride (R18). Fluorescence images are pseudocolored for better visualization. Scale bar is 10 μm.
